# Identification of Challenging Dermatophyte Species Using Matrix-Assisted Laser Desorption/Ionization Time-of-Flight Mass Spectrometry

**DOI:** 10.3390/jof11020107

**Published:** 2025-01-31

**Authors:** Tsung-Fu Tsai, Yun-Chen Fan, Jang-Jih Lu, Chun-Chih Chien, Hsin-Yao Wang, Pei-Lun Sun

**Affiliations:** 1 Department of Dermatology, Chang Gung Memorial Hospital, Linkou Branch, Taoyuan 333, Taiwan; henry@gap.kmu.edu.tw; 2 School of Medicine, College of Medicine, Chang Gung University, Taoyuan 333, Taiwan; 3 Research Laboratory of Medical Mycology, Chang Gung Memorial Hospital, Linkou Branch, Taoyuan 333, Taiwan; fan01290129@cgmh.org.tw; 4 Division of Clinical Pathology, Taipei Tzu Chi Hospital, Buddhist Tzu Chi Medical Foundation, New Taipei City 231, Taiwan; janglu45@gmail.com; 5 Department of Laboratory Medicine, Kaohsiung Chang Gung Memorial Hospital, Kaohsiung 833, Taiwan; jessica0307@cgmh.org.tw; 6 Department of Laboratory Medicine, Chang Gung Memorial Hospital, Linkou Branch, Taoyuan 333, Taiwan; 7 School of Medicine, National Tsing Hua University, Hsinchu 300, Taiwan

**Keywords:** dermatophytes, fungi, MALDI-TOF mass spectrometry, *Trichophyton*, *Nannizzia*, *Microsporum*, *Epidermophyton*

## Abstract

Matrix-assisted laser desorption/ionization time-of-flight mass spectrometry (MALDI-TOF MS) is a widely adopted technique for bacterial and yeast identification in clinical laboratories but is less frequently applied to filamentous fungi due to inconsistent performance, limitations of commercial libraries, and variability of preparation methods. This study aimed to validate the efficiency of MALDI-TOF MS-based dermatophyte identification using the Bruker Biotyper system. Focusing on species from the *Trichophyton*, *Nannizzia*, *Microsporum*, and *Epidermophyton* genera, an in-house reference library was established and evaluated with clinical isolates. The expanded library, which combined the in-house and Bruker libraries, achieved significantly higher accuracy than the Bruker library alone, correctly identifying 90.7% (107/118) of isolates at the species level compared to 16.1% (19/118) by the Bruker library. This study presents an efficient, standardized MALDI-TOF MS protocol for routine dermatophyte identification and provides a review of the current status and influencing factors in MALDI-TOF MS-based dermatophyte identification strategies.

## 1. Introduction

Dermatophytes are the key fungal pathogens for various skin infections, such as tinea capitis, corporis, and pedis. These fungi are transmitted via direct contact with infected individuals, animals, and soil as well as indirect contact with contaminated surfaces, posing a considerable healthcare burden [1]. Proper identification of dermatophytes at the species level provides reliable epidemiological data and clues to determine the infection sources for effective disease control. Traditional microscopic identification of dermatophytes relies on phenotypic techniques requiring skilled personnel that are often labor-intensive with a prolonged turnaround time [2,3]. Molecular methods, such as polymerase chain reaction and DNA sequencing, provide high accuracy and speed, but are costly, technically complex, and scarce in resource-limited settings [4]. Matrix-assisted laser desorption/ionization time-of-flight mass spectrometry (MALDI-TOF MS), initially developed as a proteomics tool, has expanded its application from bacterial to yeast identification and even filamentous fungal identification, to some extent. This technology offers advantages, such as speed, simplicity, and cost-effectiveness, facilitating rapid and high throughput identification with reliable species-level accuracy in clinical and laboratory settings [5,6,7]. The use of MALDI-TOF MS for fungal identification has yielded promising results. When used in conjunction with both commercial and in-house libraries, some studies have reported identification rates of up to 90%, surpassing those of traditional methods for distinguishing morphologically similar species, detecting drug-resistant phenotypes, and expediting the identification of microorganisms directly from positive blood cultures [8,9,10,11]. Despite these benefits, this technique has many limitations, including the need for standardized criteria, batch effects, different endemic characteristics, and limited protocol reproducibility [12,13]. Compared to the relatively simple and consistent life cycles of disease-causing bacteria, fungi exhibit a highly complex life cycle. The varying growth rates among fungi lead to the expression of different proteins. Moreover, spores, conidiophores, and mycelia may all be present in the same colony at the time of MALDI-TOF analysis, resulting in suboptimal diagnostic performance [14,15].

MALDI-TOF MS identification begins with ionization and molecular separation based on the mass-to-charge ratio (*m*/*z*). A nitrogen laser imposes energy on the molecules of lysis microorganisms embedded in an organic matrix, causing the molecules to desorb, vaporize, and ionize. These ionized molecules pass through an electric field and vacuum tube, where their velocity correlates with the *m*/*z* ratio. At the end of the vacuum tube, the detector captures the ionized molecules and measures their time-of-flight and abundance. The resulting raw spectrum is subjected to several preprocessing steps and compared with a database of known reference spectra [16,17]. Microorganism identification hinges on the similarity between the sample and best-matched reference spectra, which vary from genus to species levels [18].

MALDI-TOF MS detection of dermatophytes requires special consideration due to the challenges in phenotypically identifying these fungi, their lengthy growth periods, and underrepresentation compared to yeasts, bacteria, and other clinical filamentous fungi [19]. Commercial libraries, such as the Bruker MBT Filamentous Fungi Library, indicate that species within the *Trichophyton*, *Microsporum*, and *Nannizzia* genera exhibit very similar patterns, making it difficult to distinguish among these species using MALDI-TOF MS [20]. Additionally, reliance on commercial libraries alone is limited by their incomplete inclusion of local strains, highlighting the importance of in-house reference libraries, as shown in several studies [21,22,23]. Local strains typically have more consistent protein compositions, and incorporating their spectral profiles can improve MALDI-TOF MS identification rates. Combining both libraries can further enhance identification accuracy when the protein composition of strains in the commercial library aligns with that of unique local strains. Thus, the aim of establishing an in-house reference library is not to replace commercial libraries but to improve identification rates by diversifying spectral profiles, thereby addressing gaps in existing databases and enhancing coverage for local strains.

Here, we aimed to enhance MALDI-TOF-based dermatophyte identification by proposing a practical and reproducible protocol and evaluating the performance of an expanded library combining an in-house MS library of local isolates with the commercial Bruker MBT Filamentous Fungi Library v4.0 (Bruker Daltonics). The expanded library consistently outperformed the Bruker library, achieving identification rates of 88.0–100% for *Trichophyton* species, 100% for *Microsporum canis*, and 75.0–100% for *Nannizzia* species, at an LS cutoff of 1.7. These higher identification rates demonstrate the feasibility of the standardized protocol and highlight the critical role of an in-house library in accurately identifying challenging dermatophyte species using MALDI-TOF MS.

## 2. Materials and Methods

### 2.1. Dermatophyte Selection and Culture Conditions for the In-House Library and Test Isolates

For this study, dermatophyte isolates were selected from the culture collection of the Research Laboratory of Medical Mycology at Chang Gung Memorial Hospital, Linkou Branch, Taiwan. These isolates represented eleven species across three genera: seven *Trichophyton* (*T. interdigitale*, *T. rubrum*, *T. mentagrophytes*, *T. tonsurans*, *T. indotineae*, *T. benhamiae*, and *T. erinacei*), two *Nannizzia* (*N. gypsea* and *N. incurvata*), one *Microsporum* (*M. canis*), and one *Epidermophyton* (*E. floccosum*) species. The isolates were indigenous to Taiwan and obtained from humans, animals, or the environment. All isolates were identified to the species level based on their morphological characteristics and sequence-based methods using the internal transcribed spacer (ITS) regions of ribosomal DNA. For the construction of the reference library and testing, the isolates were revived from the culture collection, subcultured onto solid potato dextrose agar (PDA) plates (BD DIFCO™), and incubated at 25 °C for seven days prior to protein extraction. A total of 73 isolates were used to construct the in-house library, while 118 isolates were utilized to evaluate the efficiency of MALDI-TOF MS analysis. The numbers of isolates for each species used in the in-house library and for evaluation are detailed in Table 1.

### 2.2. Sample Preparation for MALDI-TOF MS and Protein Extraction

Dermatophyte proteins were extracted using a formic acid–ethanol protein extraction procedure, following the manufacturer’s recommended protocol (Standard Operating Procedure for Cultivation and Sample Preparation for Filamentous Fungi; Bruker Daltonics). A portion of the fungal mycelia was collected and suspended in 240 μL of distilled water and 900 μL of 95% ethanol. After thorough vortexing, centrifugation was performed at 13,000× *g* for 8 min, and the supernatant was extracted using a pipette tip. Subsequently, a second centrifugation was performed at 13,000× *g* for 2 min, and the samples were air-dried as necessary. The resulting pellet was resuspended in 10 μL of 70% formic acid, thoroughly mixed with a pipette, vortexed, and spun down. The samples were incubated at 25 °C room temperature for at least 5 min. After the addition of 10 μL of 100% acetonitrile, the mixture was pipette-mixed, vortexed, and spun down. The samples were kept at room temperature for at least 10 min. After a final centrifugation for 2 min at 13,000× *g*, the supernatant was collected, and 1 μL was spotted on a plate (MTP 384 target plate polished steel TF; Bruker Daltonics, Bremen, Germany). It was overlaid with 1 μL of alpha-cyano-4-hydroxycinnamic acid matrix solution (Sigma Aldrich, St. Louis, MO, USA) and air-dried before MALDI-TOF MS analysis.

### 2.3. MS Data Acquisition

MALDI-TOF MS assays were conducted using the Microflex LT mass spectrometer (Bruker Daltonics) and analyzed using the MALDI Biotyper Software (version 3.4; Bruker Daltonics) and FlexAnalysis software (version 3.4; Bruker Daltonics). Default parameters were configured accordingly (linear positive ion mode; laser frequency: 200 Hz; ion source 1 voltage: 20 kV; ion source 2 voltage: 16.7 kV; lens voltage: 7.0 kV; mass range: 2–20 kDa). Each spectrum involved the accumulation and analysis of 640 signals following the manufacturer’s procedure in the automatic or manual mode. The TOF measurements were converted to mass-to-charge values, and all raw spectra were automatically processed. The resulting peak lists were exported to MALDI Biotyper, and only peaks with signal/noise ratio ≥ 10 were considered. In each experiment, the Bruker Bacterial Test standard (Bruker Daltonics) was used for calibration and control, according to the manufacturer’s instructions.

### 2.4. Determination of the Main Spectra Profile (MSP) from Reference Isolates and Construction of the In-House Dermatophyte Library

**Figure 1:**
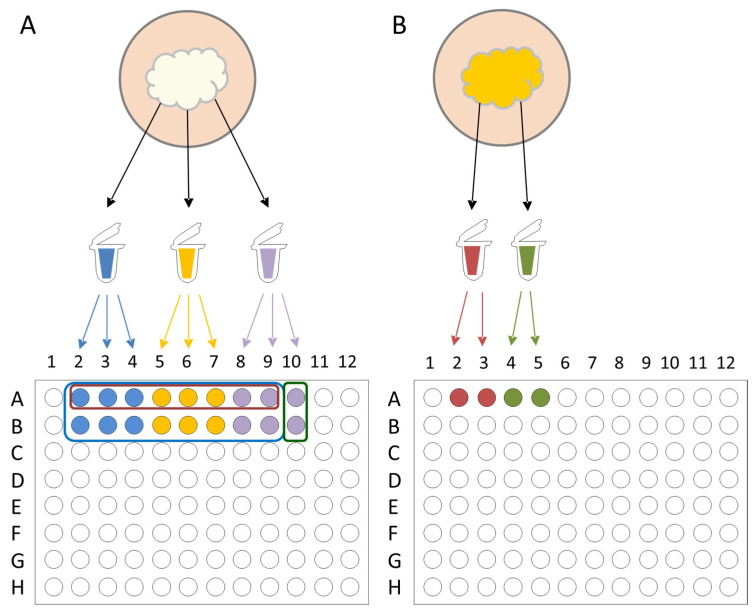
Process of spectra acquisition from the reference isolates. (**A**) Protein extracts of all isolates were prepared in triplicate and spotted onto a target plate, with six spots per extract. Automated mass spectrometry (MS) acquisition was performed for eight spots (red boxes, A2–A9), and manual acquisition was performed for 16 spots (blue boxes, A2–A9 and B2–B9), totaling 24 spectra. Two spectra (green boxes, A10 and B10) were used for within-run internal validation. (**B**) Validation: Two protein extracts from the same isolates were spotted to the plate in duplicate, yielding a total of four spots. Isolates were retained only if they achieved a log score (LS) ≥ 1.7 during matrix-assisted laser desorption/ionization time-of-flight (MALDI-TOF) identification against both the self-constructed main spectra profile (MSP) and the existing Bruker library in at least three of the four collected spectra.

The identification threshold for spectral analysis was set at a log score (LS) of 1.7, as proposed by previous studies, which demonstrated that an LS of 1.7 significantly increases the identification rate for filamentous fungi at the species level without causing substantial misidentifications [24,25]. Reference spectra were generated from the 73 dermatophyte isolates listed in Table 1, according to the manufacturer’s recommended protocol (Creation of Library Entries Tutorial, Revision A; Bruker Daltonics). Protein extracts of all isolates were prepared in triplicate and spotted onto a target plate, with six spots per extract. Automated MS acquisition was performed for 8 spots, whereas manual acquisition was performed for 16 spots, resulting in 24 spectra. Two spectra were used for within-run internal validation, and reference spectra were acquired only if both achieved a log score (LS) ≥ 1.7. FlexAnalysis software was used to generate a compilation of the most notable peaks based on the average masses, average intensities, and relative frequencies. The resulting spectra were compiled into the MSP to establish an in-house dermatophyte library. To validate this library, the isolates were applied to four spots and subjected to MALDI-TOF MS identification using the self-constructed MSP and the existing Bruker MBT Filamentous Fungi Library v4.0. Isolates meeting the cutoff LS ≥ 1.7 in at least three of the four collected spectra were retained and the remaining were excluded (Figure 1). This process was repeated until all the isolates fulfilled the requirements.

### 2.5. Validation of the Bruker and Expanded Libraries via MALDI-TOF MS and Identification of Sequence-Confirmed Test Isolates

A validation set of 118 test isolates was analyzed via MALDI-TOF MS. Each isolate was analyzed in quadruplicate, with four spots per isolate. The LS value was determined by comparing the collected spectra with those of the Bruker library alone and expanded library (consisting of the Bruker library combined with the constructed in-house library). Accurate identification was achieved when at least three of the four spots matched the reference spectra of the same species, meeting the defined cutoff LS ≥ 1.7. All identifications not meeting this criterion were considered incorrect. Results were also recorded using cutoff LS values of ≥1.8, 1.9, and 2.0 for the expanded library, and ≥2.0 for the Bruker library.

### 2.6. Statistical Analysis

Statistical analysis was performed to evaluate identification accuracies, calculated as the percentage of correctly identified isolates at the species level for each library, based on predefined cutoff log scores as previously described. Differences in identification rates between the Bruker and expanded libraries were assessed using chi-square tests when all expected frequencies were ≥5. For species with small sample sizes or expected frequencies <5, Fisher’s exact test was used as an alternative. A *p*-value of <0.05 was considered statistically significant. All analyses were conducted using SPSS software (version 24.0; IBM, Armonk, NY, USA).

## 3. Results

**Figure 2:**
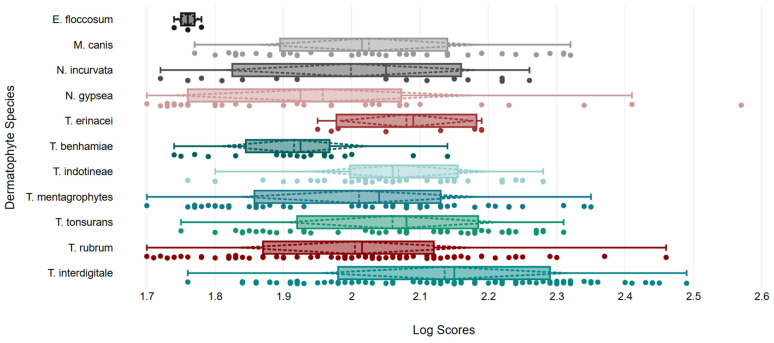
Identification performance of the expanded library (cutoff LS: 1.7). Box plot illustrating the identification performance of the expanded library with the distribution of LS values of extracts from identified dermatophyte isolates. Each colored box represents the distribution of LS values for a specific species, with individual data points plotted as dots. The central line within each box plot indicates the median LS, and edges of the box indicate the interquartile range. Whiskers extend to one standard deviation above and below the mean of the data. This figure was created using DATAtab (DATAtab Team, 2024) [26].

The identification efficiencies of the Bruker and expanded library were evaluated using 118 dermatophyte isolates, including *Trichophyton* (n = 91), *Nannizzia* (n = 16), *Microsporum* (n = 10), and *Epidermophyton* (n = 1). The expanded library consistently outperformed the Bruker library across all species. For *Trichophyton*, the Bruker library exhibited low identification rates at an LS cutoff of 1.7, ranging from 0% for *T. mentagrophytes*, *T. indotineae*, *T. benhamiae*, and *T. erinacei* to 23.8% for *T. rubrum*. In contrast, the expanded library identified 88.0–100% of isolates at an LS cutoff of 1.7, with rates declining at higher cutoffs (1.8, 1.9, and 2.0). For *Nannizzia*, the expanded library achieved superior rates of 75.0% for *N. gypsea* and 100.0% for *N. incurvata*, compared to 8.3% and 0% using the Bruker library at an LS cutoff of 1.7. Similar trends of reduced accuracy were observed at higher LS cutoffs. For *M. canis*, the Bruker library identified 80.0% of isolates, while the expanded library achieved 100% at an LS cutoff of 1.7, declining to 50.0% at an LS cutoff of 2.0. For *E. floccosum*, both libraries correctly identified the single isolate at LS 1.7 but failed at higher cutoffs. Statistical analysis revealed that, at an LS cutoff of 1.7, the expanded library demonstrated significantly better performance for all *Trichophyton* and *Nannizzia* species, except for *T. erinacei*. For *M. canis*, both libraries showed high identification rates with no significant difference (*p* = 0.474) at an LS cutoff of 1.7. At an LS cutoff of 2.0, the expanded library continued to exhibit significantly superior performance for *T. interdigitale*, *T. indotineae*, *T. mentagrophytes*, *T. tonsurans*, and *T. rubrum*. Detailed identification rates at various LS cutoffs, along with *p*-values for comparisons between the libraries at LS cutoffs of 1.7 and 2.0, are provided in Table 2. Additionally, the distribution of LS scores for correctly identified strains using the expanded library at an LS cutoff of 1.7 is shown in Figure 2.

## 4. Discussion

Currently, MALDI-TOF MS is routinely used in clinical laboratories for the identification of bacteria and yeast. Additionally, it shows potential as a promising, rapid, and cost-effective tool for the identification of filamentous fungi. Various commercial MALDI-TOF MS systems, such as Vitek MS, Saramis, Andromas, and MicroIDSys Elite, are equipped with fungal software modules and reference databases for yeast and filamentous fungal identification [27]. Among these, the Bruker Biotyper platform, along with its commercial identification library, is the most used system to date [25,28]. In this study, we used four groups of sequence-verified dermatophytes to establish an in-house MS library for validating MALDI-TOF MS identification with the Bruker Biotyper system.

Species-level identification of these dermatophytes using the Bruker library alone was unsatisfactory, particularly for *Trichophyton* and *Nannizzia* species, with identification rates ranging from 0% to 23.8%. However, these rates substantially improved with the inclusion of our in-house library. The first group of dermatophytes included the *Trichophyton* species, *T. interdigitale*, *T. rubrum*, *T. mentagrophytes*, *T. tonsurans*, *T. indotineae*, *T. benhamiae*, and *T. erinacei*. With the addition of the in-house library, genus-level identification was achieved for all tested isolates, with species-level identification rates ranging from 88.0% to 100%. Accurate identification of *Trichophyton* species is crucial for both epidemiological studies and effective treatment strategies. Notably, host specificity in zoophilic *Trichophyton* species, such as *T. benhamiae* (guinea pigs and rabbits), *T. erinacei* (hedgehogs), and *T. mentagrophytes* (rabbits and rats), allows for tracing infection sources [29,30,31]. Furthermore, distinguishing between species such as *T. interdigitale*, *T. tonsurans*, and *T. indotineae*—which share morphological features with *T. mentagrophytes*—is essential. The high identification accuracy (90.9%) of *T. indotineae*, an emerging terbinafine-resistant pathogen, emphasizes the utility of MALDI-TOF MS in screening for drug-resistant dermatophytes [32].

The second group consisted of two species of *Nannizzia*: *N. gypsea* and *N. incurvata*. Differentiating these species based on colony and microscopic features is challenging and often requires additional methods, such as mating tests and molecular techniques like DNA sequencing [33,34]. Considering the variations in MALDI-TOF MS spectra, we hypothesized that MALDI-TOF MS can effectively differentiate among these dermatophyte species. Using the expanded library, all four *N. incurvata* isolates were successfully identified with 100% accuracy, while the identification rate for the 12 *N. gypsea* test isolates was 75.0%, significantly higher than the 8.3% rate observed with the Bruker library.

*M. canis*, the most common zoonotic dermatophyte, requires accurate identification for effective treatment and infection control [2]. An important differential diagnosis is *M. audouinii*. Although *M. audouinii* was absent from the in-house library due to the unavailability of local isolates, *M. canis* was identified with 100% accuracy using MALDI-TOF MS, without any misidentifications. Furthermore, *E. floccosum*, an anthropophilic dermatophyte, was accurately identified with both libraries, primarily due to the MSP from the Bruker library.

A major challenge in MALDI-TOF MS identification of dermatophytes is overcoming the limitations of reference spectra in commercial and open-access databases. In fact, all studies evaluating this method for dermatophyte identification have relied on in-house reference libraries [19]. Additionally, the success of species identification is closely tied to the number of strain entries in the reference spectra library for each species [8,35,36]. For example, Karabic¸ak et al. achieved a significantly higher identification rate of 96.8% for 126 dermatophyte isolates when supplementing with an in-house library, compared to only 51.6% using the Bruker library alone [37]. Similarly, Maldonado et al. showed that adding an in-house library to the Bruker library improved species-level MALDI-TOF MS identification of 136 dermatophyte isolates, increasing the success rate from 45% to 88% [38]. Moreover, Arnaud Jabet et al. enhanced species-level identification from 63.4% to 91.7% for 111 molecularly identified dermatophyte strains by enriching the Mass Spectrometry Identification database-2 (MSI-2) with additional spectra obtained from cultures grown on two different media at various culture ages [39]. Our results also support the view that supplementing spectra from regional isolates is crucial for improving dermatophyte identification using MALDI-TOF MS.

In addition to the limitations of available reference spectra, several other challenges arise when applying MALDI-TOF MS for the identification of filamentous fungi, as it is more complex than for other microorganisms [23]. These challenges include the inherent difficulty of obtaining high-quality protein extracts from mycelia with diverse morphotypes or structural characteristics, variations in protein extraction methods, differences in fungal growth periods, variability in the construction of reference spectral methodologies, differing interpretive criteria in the literature, and manufacturer-established scores for some species [40,41,42]. These factors contribute to the variability in the reproducibility of MALDI-TOF MS-based dermatophyte identification.

The Bruker library was initially established using a labor-intensive liquid culture method with a formic acid–ethanol extraction protocol. However, this process of obtaining mycelia is impractical for routine clinical settings. The literature has addressed this limitation with more practical approaches, including various protein extraction methods and alternative culture techniques.

The extended direct transfer method, in which the mycelium is directly overlaid with a matrix solution prior to MALDI-TOF MS analysis, exhibits inconsistent performance across studies. Some reports have suggested that this method yields suboptimal identification rates, especially for closely related species or rare isolates, due to insufficient protein extraction or ionization [24,43]. Conversely, other studies indicate that, under optimized conditions, the direct transfer method can achieve identification rates comparable to those of the more labor-intensive formic acid–ethanol extraction method, making it a faster alternative for routine clinical use [44,45]. Currently, the formic acid–ethanol extraction method remains the preferred protocol in many laboratories due to its consistent and reliable performance for various fungal species, facilitating efficient protein extraction with enhanced signal quality [8,46,47].

Many laboratories adopt solid culture methods to obtain mycelium. Interestingly, some studies have suggested that selective culture media, such as the Sabouraud chloramphenicol gentamicin agar or ID fungi plate, outperform non-selective media, such as the Sabouraud dextrose agar or PDA, in MALDI-TOF MS identification of filamentous fungi [42,48]. Nevertheless, liquid cultivation remains the most reliable approach for identification using the Bruker library, particularly in cases where other methods fail to deliver accurate results. In this study, we selected a solid culture PDA and formic acid–ethanol protein extraction procedure, as previously outlined, with a standardized fungal growth period of seven days.

To determine the degree of similarity between the test specimen and reference sample, cutoff LS was used. According to Bruker recommendations, LS  >  2.3 suggests highly probable species identification, 2 < LS  <  2.299 indicates secure genus identification and probable species identification, 1.7 < LS  <  1.999 indicates probable genus identification, and LS  <  1.7 indicates unreliable identification [25]. However, these score thresholds were designed for bacterial identification and may not be suitable for fungi, especially molds and dermatophytes. Schulthess et al. compared the 1.7 and 2.0 score thresholds for MALDI-TOF MS identification after liquid cultivation and revealed that a species cutoff of 1.7 was highly reliable for mold identification using the Filamentous Fungi Library v1.0 [49]. In a study by Zvezdanova et al., identification rate of filamentous fungi by MALDI-TOF MS at an LS cutoff of 1.8 was identical to that of DNA sequencing analysis in 96.7% of cases [22]. Some studies have also indicated species correlation with LS values below 1.6, highlighting the distinctions in bacterial and fungal identification using MALDI-TOF MS [50]. To date, most publications have used a single score threshold, generally ranging from 1.7 to 2.0. In this study, LS of 1.7 excellently correlated with 99.1% (106/107) sequence identification. However, our expanded library misidentified one isolate of *T. interdigitale* as *T. indontineae*, underscoring the possible overlapping species-specific protein patterns within these groups.

The number of samples analyzed for identification varies among different studies, with some using duplicate or quadruplicate analyses [51]. Increasing the number of samples mitigates the errors due to potential variability within the same strain [34]. In this study, protein extraction from fungal isolates was performed in quadruplicate. This approach aligns with the proposal of Cassagne et al., who indicated that at least three out of four spots from a clinical isolate should match MSPs of the same species to be considered concordant and interpretable [52].

Stein et al. comparatively analyzed three mass spectral libraries to identify filamentous fungi [53]. Using the Bruker system, they assessed 63 reference and 158 local clinical isolates from various sample types, generating spectra that were analyzed using the MSI 2016 library, Bruker Filamentous Fungi Library v1.0, and NIH library database. The MSI library exhibited the highest rate of species-level identification (72.0%), surpassing those of the NIH (19.5%) and Bruker (13.6%) libraries. However, over 20% of the molds could not be identified to the species level using any of the tested MALDI-TOF MS libraries, primarily because of library limitations and insufficient spectra. Considering the strengths and limitations of different libraries, some studies recommend using datasets together, when possible, for optimal fungal identification [42]. The significant variability in MALDI-TOF MS fungal identification across different libraries once again underscores the need for an in-house library for each clinical laboratory, as these resources overcome the limitations of available databases and are especially useful in regions with endemic or underrepresented fungal strains [23,24,52].

## 5. Conclusions

Proper differentiation of dermatophytes using conventional methods is challenging owing to their morphological similarities, lack of well-defined species within complexes, and taxonomic proximity [54]. MALDI-TOF MS is a promising and rapidly advancing technique for filamentous fungal identification. Previous studies have demonstrated its capacity for the rapid and accurate identification of common dermatophytes, closely aligned with ITS sequencing results, better than traditional phenotypic methods [55,56,57]. In this study, we proposed and validated a practical and reproducible 7-day protocol for MALDI-TOF MS-based dermatophyte identification. We also demonstrated the superior performance of an expanded library compared to that of a commercial library alone in distinguishing difficult-to-identify species within the *Trichophyton*, *Microsporum*, and *Nannizzia* genera. These findings support the routine use of MALDI-TOF MS for dermatophyte identification in clinical laboratories.

## Figures and Tables

**Table 1:** Number of isolates used to construct the in-house library and number of test isolates.

Dermatophyte Species	Number of Isolates Used to Construct the In-House Library	Number of Test Isolates
*Trichophyton interdigitale*	15	25
*Trichophyton rubrum*	20	21
*Trichophyton mentagrophytes*	5	15
*Trichophyton tonsurans*	4	12
*Trichophyton indotineae*	4	11
*Trichophyton benhamiae*	3	5
*Trichophyton erinacei*	3	2
*Nannizzia gypsea*	7	12
*Nannizzia incurvata*	7	4
*Microsporum canis*	3	10
*Epidermophyton floccosum*	2	1
Total	73	118

**Table 2:** Identification of the 118 test isolates using the Bruker and expanded libraries at different cutoff log score values.

		Correct Identification: Number (Percentage)						*p*-Value	
		Bruker		Expanded (Bruker + In-House)				Cutoff: 1.7	Cutoff: 2.0
Dermatophyte Species	Number of Test Strains	Cutoff: 1.7	Cutoff: 2.0	Cutoff: 1.7	Cutoff: 1.8	Cutoff: 1.9	Cutoff: 2.0		
*Trichophyton* *interdigitale*	25	2 (8%)	0 (0%)	22 (88%)	22 (88%)	19 (76%)	15 (60%)	<0.001	<0.001
*Trichophyton* *indotineae*	11	0 (0%)	0 (0%)	10 (90.9%)	10 (90.9%)	7 (63.6%)	6 (54.5%)	<0.001	0.012
*Trichophyton* *mentagrophytes*	15	0 (0%)	0 (0%)	14 (93.3%)	10 (66.7%)	7 (46.7%)	7 (46.7%)	<0.001	<0.001
*Trichophyton* *tonsurans*	12	2 (16.7%)	0 (0%)	11 (91.7%)	11 (91.7%)	8 (66.7%)	5 (41.7%)	<0.001	<0.001
*Trichophyton* *benhamiae*	5	0 (0%)	0 (0%)	5 (100%)	3 (60%)	3 (60%)	0 (0%)	0.008	1.0
*Trichophyton* *erinacei*	2	0 (0%)	0 (0%)	2 (100%)	2 (100%)	2 (100%)	2 (100%)	0.333	0.333
*Trichophyton* *rubrum*	21	5 (23.8%)	0 (0%)	19 (90.5%)	17 (81%)	11 (53.4%)	10 (47.6%)	<0.001	<0.001
*Microsporum* *canis*	10	8 (80%)	3 (30%)	10 (100%)	10 (100%)	7 (70%)	5 (50%)	0.474	0.650
*Nannizzia* *gypsea*	12	1 (8.3%)	0 (0%)	9 (75%)	5 (41.7%)	3 (25%)	2 (16.7%)	<0.001	0.478
*Nannizzia* *incurvata*	4	0 (0%)	0 (0%)	4 (100%)	2 (50%)	2 (50%)	2 (50%)	0.029	0.429
*Epidermophyton* *floccosum*	1	1 (100%)	0 (0%)	1 (100%)	0 (0%)	0 (0%)	0 (0%)	1.0	1.0

## Data Availability

The data that support the findings of this study are available from the corresponding author, Pei-Lun Sun, M.D., Ph.D., upon reasonable request.

## Abbreviations

The following abbreviations are used in this manuscript:

ITS	Internal transcribed spacer
LS	Log score
m/z	Mass-to-charge ratio
MALDI-TOF MS	Matrix-assisted laser desorption/ionization time-of-flight mass spectrometry
MS	Mass spectrometry
MSI	Mass Spectrometry Identification database
MSP	Main spectra profile
PDA	Potato dextrose agar
